# Toward systematic reviews to understand the determinants of wait time management success to help decision-makers and managers better manage wait times

**DOI:** 10.1186/1748-5908-8-61

**Published:** 2013-06-06

**Authors:** Marie-Pascale Pomey, Pierre-Gerlier Forest, Claudia Sanmartin, Carolyn DeCoster, Nathalie Clavel, Elaine Warren, Madeleine Drew, Tom Noseworthy

**Affiliations:** 1 Department of Health Administration, Institut de Recherche en Santé Publique de l’Université de Montréal (IRSPUM), University of Montreal, 7101 Parc Avenue, Montreal, Quebec, H3N 1X7, Canada; 2 Pierre Elliott Trudeau Foundation, 1514 Docteur-Penfield Avenue, Montreal, Quebec, H3G 1B9, Canada; 3 Health Analysis Division, Statistics Canada, 150 Tunney’s Pasture Driveway, Ottawa, Ontario, K1A 0T6, Canada; 4 Data Integration, Measurement & Reporting Service, Alberta Health Services, 10101 Southport Road SW, Calgary, Alberta, T2W 3N2, Canada; 5 Surgical Services, Eastern Health, Health Sciences Centre, Prince Philip Drive, St. John’s, Newfoundland, A1B 3V6, Canada; 6 Accreditation Canada, 1150 Cyrville Road, Ottawa, Ontario, K1J 7S9, Canada; 7 Department of Community Health Sciences, University of Calgary, 2500 University Drive NW, Calgary, Alberta, T2N 1N4, Canada

**Keywords:** Wait times, Management strategies, Scheduled care, Implementation and Sustainability factors

## Abstract

**Background:**

Long waits for core specialized services have consistently been identified as a key barrier to access. Governments and organizations at all levels have responded with strategies for better wait list management. While these initiatives are promising, insufficient attention has been paid to factors influencing the implementation and sustainability of wait time management strategies (WTMS) implemented at the organizational level.

**Methods:**

A systematic review was conducted using the main electronic databases, such as CINAHL, MEDLINE, and Cochrane Database of Systematic Reviews, to identify articles published between 1990 and 2011 on WTMS for scheduled care implemented at the organizational level or higher and on frameworks for analyzing factors influencing their success. Data was extracted on governance, culture, resources, and tools. We organized a workshop with Canadian healthcare policy-makers and managers to compare our initial findings with their experience.

**Results:**

Our systematic review included 47 articles: 36 related to implementation and 11 to sustainability. From these, we identified a variety of WTMS initiated at the organizational level or higher, and within these, certain factors that were specific to either implementation or sustainability and others common to both. The main common factors influencing success at the contextual level were stakeholder engagement and strong funding, and at the organizational level, physician involvement, human resources capacity, and information management systems. Specific factors for successful implementation at the contextual level were consultation with front-line actors and common standards and guidelines, and at the organizational level, financial incentives and dedicated staffing. For sustainability, we found no new factors. The workshop participants identified the same major factors as found in the articles and added others, such as information sharing between physicians and managers.

**Conclusions:**

Factors related to implementation were studied more than those related to sustainability. However, this finding was useful in developing a tool to help managers at the local level monitor the implementation of WTMS and highlighted the need for more research on specific factors for sustainability and to assess the unintended consequences of introducing WTMS in healthcare organizations.

## Background

For the past two decades, access to healthcare services has been a critical issue in most Organisation for Economic Co-operation and Development (OECD) countries [1]. Long waits for core specialized healthcare services have consistently been identified as a key barrier to access to care [2,3], and governments and organizations have responded with a range of strategies to better manage wait times. For example, in September 2004, Canada’s First Ministers committed $5.5 billion to timely access in five healthcare areas over a 10-year period [4]. In June 2005, the Supreme Court of Canada (in the Chaoulli Decision) struck down Quebec’s ban on private insurance for Medicare-covered services in a bid to reduce wait times in that province. In April 2007, Canada’s federal government announced it would provide $612 billion to provinces that would commit to respecting maximum wait times for at least one medical procedure in their jurisdiction [5]. From these initiatives, it is clear that over the past decade Canadian decision-makers have consistently seen the centralization of programs at the federal and provincial levels as the means to solve problems regarding wait lists and wait times [6]. While these initiatives are promising [7-9], we believe insufficient attention has been paid to how healthcare organizations themselves have implemented strategies to reduce wait lists and wait times [10]. We therefore conducted a systematic review, supplemented by a workshop with experts, to better understand the key organizational and contextual level factors that influence the success or failure of wait time management strategies (WTMS) undertaken by organizations, not only in their implementation but also in their sustainability over the longer term. Given that WTMS implementation and sustainability have not been defined in the literature as such, we borrowed from the change theory literature on the life cycle of typical change initiatives [11], where the implementation phase is defined as the period when a new intervention is introduced and the sustainability phase as the period during which it is consolidated. In the systematic review and expert workshop, we focused on factors that were considered to influence the success of WTMS implementation and sustainability; we did not focus directly on the impact of these factors on reducing wait times. Our systematic review explored five questions:

1. Are there existing frameworks to identify potential explanatory factors for WTMS success at the organizational level?

2. What contextual and organizational factors can influence the success of WTMS implementation at the organizational level for scheduled care?

3. What contextual and organizational factors can influence the success of WTMS sustainability at the organizational level for scheduled care?

4. Are those factors valid for the Canadian context?

5. How can identifying such factors contribute to developing a tool to support managers in implementing and sustaining WTMS at the organizational level?

## Methods

The systematic review was limited to published articles. Six electronic medical databases were searched: CINAHL, the Cochrane Central Register of Controlled Trials, the Cochrane Database of Systematic Reviews, Embase, Healthstar, and MEDLINE (including MEDLINE In-Process). We consulted 19 electronic non-medical databases, which included general Canadian databases (Canadian Business & Current Affairs; Canadian Newsstand; Canadian Periodical Index; Canadian Research Index; Repères), math and engineering databases (Compendex; IEEE Xplore; Inspec; MathSciNet), economics databases (EconLit & ERIC [CBSCO Host]), and sociological (Communications Abstracts; ERIC; PAIS; Social Sciences Abstracts; Sociological Abstracts) and multidisciplinary (ABI Inform; ProQuest Dissertations & Theses; Scopus) databases. We used the search terms ‘waiting lists’ [or] ‘wait time’ [or] ‘queues’, combined using the Boolean operator and with terms reflecting management issues, such as ‘policies’, ‘information systems’, ‘budgets’, ‘health priorities’, ‘patient referral’, ‘health care delivery’, and ‘personnel management’. Additional search strategies to complement the search outlined above were developed for both medical and non-medical databases to ensure that we captured all relevant articles on WTMS. One example of the search strategies used is given in Table 1. This search strategy was developed and used for the Cochrane Central Registry of Controlled Trials, Healthstar, and MEDLINE medical databases.

**Table 1:** **Cochrane Central Register of Controlled Trials (OVID 4**^**th **^**Quarter 2005), Healthstar (OVID 1966 to present) and MEDLINE (OVID 1966 to present) search terms strategy**

	
1.	Waiting lists [Mesh Subject exploded]
2.	Wait* time* or waitlist* or queue* [*Text words]
3.	(wait* or await*) ADJ2 (list or lists or time*)[*Text words]
4.	Awaiting [Title Word]
5.	1 or 2 or 3 or 4
6.	Transplantation or transplants or tissue donors or emergencies or emergency medical services or emergency service, hospital or emergency service, psychiatric [MeSH Major Subject exploded]
7.	5 not 6
8.	Limit 7 to yr = ‘1990 – 2006’
9.	Limit 8 to abstracts
10.	Limit 8 to English language
11.	9 or 10
12.	National health programs or local government [MeSH Subjects exploded]
13.	Health services accessibility / organization & administration, legislation & jurisprudence, standards [MeSH Major Subject exploded]
14.	Health policy or social control policies or organizational policy or public policy or public opinion [MeSH Major Subject exploded]
15.	Health care rationing / organization & administration, legislation & jurisprudence [MeSH Major Subject exploded]
16.	Health priorities / organization & administration, legislation & jurisprudence [MeSH Major Subject exploded]
17.	Management information systems [MeSH Major Subject exploded]
18.	Health personnel / organization & administration [MeSH Major Subject exploded]
19.	Personnel management / organization & administration, legislation & jurisprudence, manpower [MeSH Major Subject exploded]
20.	Information systems / methods, organization & administration, legislation & jurisprudence, manpower [MeSH Major Subject exploded]
21.	Organizational policy or organizational innovation or efficiency, organizational or decision making, organizational or organizational objectives models, organizational or organizational culture [MeSH Major Subject exploded]
22.	Regional health planning or regional medical programs [MeSH Subjects exploded]
23.	Delivery of healthcare / organization & administration, economics, legislation & jurisprudence [MeSH Major Subject exploded]
24.	‘Organization and administration’ / economics, organization & administration, education, legislation and jurisprudence, methods [MeSH Major Subject exploded]
25.	Decision making or budgets or systems analysis or operations research [MeSH Major Subject exploded]
26.	Quality of healthcare / organization & administration, economics, legislation & jurisprudence, methods [MeSH Major Subject exploded]
27.	Economics [MeSH Major Subject]
28.	Healthcare costs or costs and cost analysis or economics, medical [MeSH Major Subject exploded]
29.	State medicine / organization & administration, economics, standards, legislation & jurisprudence, manpower [MeSH Major Subject exploded]
30.	Resource allocation / organization & administration, economics, standards, supply & distribution, legislation & jurisprudence, manpower, methods [MeSH Major Subject exploded]
31.	Models, theoretical or computing methodologies or communication or Markov chains [MeSH Major Subject exploded]
32.	Professional practice / methods, organization & administration, education, standards, ethics, legislation & jurisprudence, manpower [MeSH Major Subject exploded]
33.	12 or 13 or 14 or 15 or 16 or 17 or 18 or 19 or 20 or 21 or 22 or 23 or 24 or 25 or 26 or 27 or 28 or 29 or 30 or 31 or 32
34.	11 and 32

The systematic review was carried out over two periods of time. The first search, conducted in 2005, covered articles published between 1990 and 2005, and the second search, conducted in 2011, covered those published between 2006 and 2011. We conducted a workshop in March 2009 with healthcare experts to validate the first findings of the systematic literature review and to ensure they were robust. We conducted our review in two steps because most of the articles retrieved in the first period were related to WTMS implementation, but workshop participants felt it would be important also to include articles related to WTMS sustainability. Consequently, we extended our research until 2011 to capture such articles. We also explored together how the factors identified could be developed into best practices to create a tool for managers in healthcare organizations responsible for WTMS. Participants were selected because they had responsibility for healthcare provision, health system leadership, and wait time management, were in a position to provide experience-based critiques, and could potentially apply the research findings in their context.

**Figure 1:**
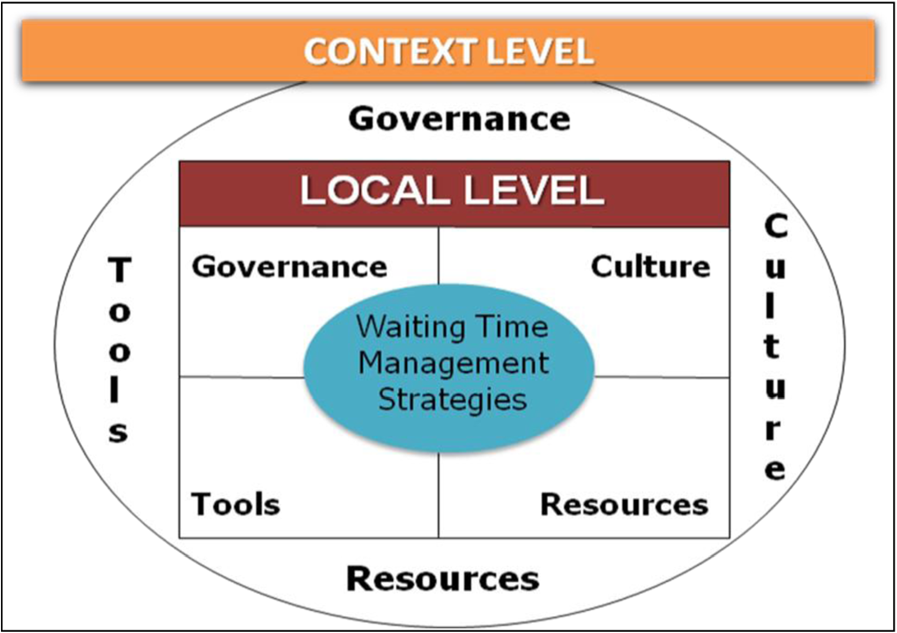
Conceptual framework.

The data from the systematic review are presented here in narrative form because of the heterogeneity of study designs in the articles examined for our systematic review. The articles reviewed used a wide variety of study designs and approaches, including case studies, pre-post comparisons studies, prospective studies, simulation/modeling studies, and time series studies. We combined the findings of the two periods, specifically identifying factors that emerged in relation to WTMS implementation or sustainability. We adapted Parson’s social system action theory to classify the factors [12]. This framework, which we used in previous work to identify contextual and organizational factors [13], was also useful to identify *a priori* coding categories [14], which is helpful when conducting reviews of qualitative studies. This framework (Figure 1) is composed of different factors that can be present at the contextual and organizational levels: ‘governance and leadership’, defined as ‘the conduct of collective action from a position of authority’ [15]; ‘culture’, which consists of ‘underlying beliefs, values, norms and behaviours’ [13,16]; ‘resources’, which may be human, financial, infrastructural, or informational; and ‘tools’, referring to instruments or procedures. The organizational level corresponds to the specific context of WTMS initiatives within healthcare organizations, whereas the contextual level refers to the broader context of WTMS initiatives, such as policies, laws, or regulations from provincial, regional, or other health authorities.

### Inclusion criteria

We applied the following four inclusion criteria:We had no inclusion criteria related to study design; all study designs were included.

1. Articles referring to wait time management for scheduled care: diagnostic imaging and elective surgery were selected for additional searches to complement the main search and to ensure all relevant articles were captured. More specifically, we selected articles that: described a model or framework of factors influencing WTMS success or failure at the organizational level; referred to an organizational initiative that specifically addressed wait time or wait lists; referred to higher level (national or provincial) strategies or policies that addressed WTMS;

2. Articles published in peer-reviewed journals;

3. Articles published in French or in English;

4. Articles published between 1990 and 2011.

### Exclusion criteria

Articles that focused on transplantation, emergency care, and psychiatric care were excluded because their wait time management differs significantly from that for scheduled care. We also excluded opinion papers and papers with no mention of the study design.

### Data extraction

Data extraction was qualitative and was performed by five members of the research team using SRS (TrialStat). The software monitored the Kappa data extraction from the five extractors, and discussions among extractors were regularly organized if necessary to reduce any inconsistency between reviewers. In addition, the principal investigator reviewed all abstracted data. The extraction form was a custom-designed template that had been tested on a sample of articles. The following main categories of data were extracted: jurisdiction of WTMS, clinical area of WTMS, definition of wait time or wait list, description of WTMS, article objectives, study design, theoretical framework used, and factors influencing WTMS (categories of factors and their positive or negative influence on WTMS). Because the links between the factors and the success of the WTMS strategies were not systematically analyzed in the articles or quantified in any heterogeneous way, we decided not to include such analyses. Whenever possible, reviewers used quotes to describe wait time initiatives, and for all factors identified in the articles, added page and paragraph references.

A search protocol for the systematic review was approved by the Canadian Institutes of Health Research and is available through the principal investigator.

## Results

### Systematic review

**Figure 2:**
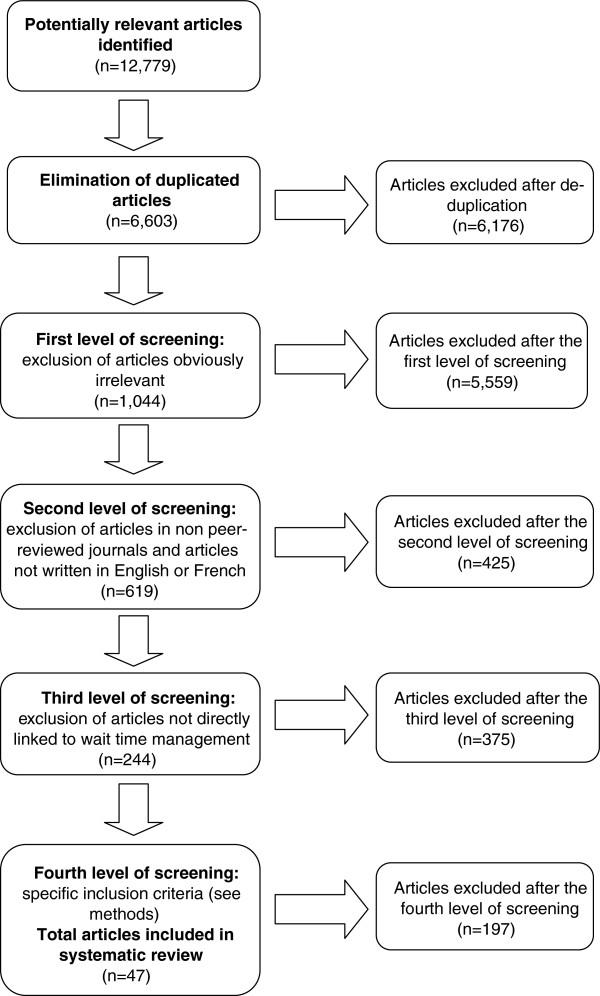
Flow diagram of the search and selection process.

The search yielded 12,779 references; after elimination of duplicates, 6,176 remained. Four levels of screening were conducted in duplicate by two reviewers using the web-based systematic reviewing platform SRS (TrialSat). The first level excluded all articles obviously irrelevant based on abstracts. The second level excluded articles in non-peer reviewed journals and those written in languages other than English and French. With 619 references remaining, the third level consisted of reviewing the titles and abstracts with the same criteria used in the second level, this time excluding articles not directly linked to wait time management. For the remaining 244 full articles, in the fourth level of screening, the research team applied the specific inclusion criteria for articles described above. In the end, 47 articles were retained and abstracted (Figure 2).

### Types of research designs and countries of provenance

Of these 47 articles, the vast majority (n = 45) were empirical studies, and the remaining two used a theoretical approach. Approximately one-third of the empirical studies were single case studies (n = 17), some were pre-/post-strategy comparisons (n = 9), others combined a case study with a survey (n = 7), some were multiple case studies (n = 6), a few were simulation/modelling (n = 3), and the rest were time series (n = 3). Two-thirds of these 47 articles were from Canada (n = 15) and the United Kingdom (n = 15). The rest were from Australia (n = 4), Sweden (n = 4), the Scandinavian countries (n = 4), New Zealand (n = 3), and the United States (n = 2). One explanation for the low number of articles from the US might be that the country faced fewer challenges regarding wait times than did other OECD countries.

### Characteristics of wait time management strategies

From the systematic review, we identified different types of WTMS in terms of the levels at which they were launched, *i.e*., at the local level in a healthcare organization or at a broader system level (national, provincial, or regional). At the local level, strategies included work reorganizations (n = 8), increases in capacity (n = 2), implementation of pre-operative clinics (n = 2), and development of data collection software, including simulations of wait lists (n = 3) and booking systems (n = 1). At the national level, strategies included booking systems (n = 6), maximum wait time guarantees (n = 4), and development and use of prioritization tools (n = 11).

Table 2 summarizes the different WTMS identified in the articles. These covered a broad range of clinical areas, including orthopaedics (n = 7), eye care (n = 7), and cardiac care (n = 6), and many covered general surgery (n = 23) or spanned multiple specialties (n = 4). Our search uncovered no studies in medical imaging, even though this area was specifically included in our research question, as wait times in this area have been identified as a problem.

**Table 2:** Types of wait time strategies reviewed in the articles according to phase of change and level of introduction

**Phase of change**	**Level of introduction**	**Strategies**	**Article numbers**
**Implementation**	***Contextual level***	Booking systems	[17,18,32,35,40]
		Maximum wait time guarantee	[33,34,37,52]
		Software development for WTM (includes simulation)	[29]
		Increases in capacity	[48]
		Pooled wait lists	[38]
		Standards or prioritization tools	[22,42,44]
		Improved data collection or data analysis	[45]
		Other (sending patients abroad, GP fund-holding, GP referral system)	[30,39,56]
	***Local level***	Increases in capacity	[43,49]
		Work reorganizations	[19,28,47,50,54]
		Pre-operatory clinic	[46,51]
		Pooled wait lists	[36]
		Standards or prioritization tools	[41]
		Improved data collection or data analysis	[55]
		Software development for WTM (includes simulation)	[20,21,29,31]
**Sustainability**	***Contextual level***	Standards or prioritization tools	[22-26,57,61-63]
	***Local level***	Work reorganizations at the local level	[54,58,60]
		Booking system	[59]

### Frameworks on factors that can influence WTMS

Among all articles abstracted, only two, by the same authors, offered a framework for studying factors that influenced the implementation of WTMS, in this case the implementation of a booking system [17,18]. Published in 2003, the study collected and analyzed quantitative and qualitative data from 20 pilot organizations in the United Kingdom to identify essential conditions for successful implementation of quality improvement initiatives in healthcare. The framework grouped the factors under six categories. Two of the categories, ‘national context’ and ‘local context’, acknowledged the need to look at the system on more than one level. This is also acknowledged in our model/framework. The authors also identified the categories ‘culture’ and ‘capacity’, which we have named ‘culture’ and ‘resources’. Another of their categories is ‘roles of physicians’, which, as in our study, emphasizes doctors’ key role. Lastly, their category ‘mechanisms of change’ covers a collection of factors, all of which have been classified in various dimensions in our own model.

### Empirical factors influencing WTMS

In the following sections, we begin by considering the factors associated with implementation at the local and contextual levels before presenting the factors associated with WTMS sustainability. A synthesis of all the factors is presented in Table 3.

**Table 3:** Factors influencing WTMS according to phase of change and level of introduction, identified from the systematic review

	**Implementation**		**Sustainability**	
	**Local level**	**Contextual level**	**Local level**	**Contextual level**
**Governance**	▪ Leadership [17-27]	▪ Reporting and monitoring structures [17,21 23-27,32,35,37,44,54]	▪ Clear accountabilities [22,42,57,58]	▪ Leadership [22,24,57,58,62]
	▪ Dedicated decision-making and management structure [19,20,24,28,29]	▪ Stakeholder engagement [22-27,32,37,54,56]	▪ Leadership [22,24,57]	▪ Stakeholder engagement [22,27,62]
	▪ Accountability (local agreement) [22,23,25,28]	▪ Leadership [23-27]		▪ Quality and safety measures, processes and monitoring [54,57]
		▪ Accountability agreement [22-25,44,54]		
**Culture**	▪ Physician involvement [17,18,26,30,32-39]	▪ Consultation with front-line actors [17,22-27,32,34,36,37]	▪ Physicians involvement [22,54]	▪ Public involvement [22,27]
	▪ Quality improvement [24-28]	▪ Public awareness and empowerment [22,23,25,39,54]	▪ Interprofessional cooperation [22]	
	▪ Trust [17,20,22,25,40]	▪ Culture of performance [22-27,54]		
**Resources**	▪ Increase capacity [17,18,20,31,37,41-44]	▪ Funding (level and earmarked resources) [17-19,22-27,30,32,34,35,37,40],[45,48,49,52,54]	▪ Appropriate capacity (human resources) [24]	▪ Funding levels [27,62]
	▪ Dedicated staffing [17,19,20,22-26,31,41,42,44-51]	▪ Financial incentives [22-26]	▪ Innovative roles for health professionals [24]	▪ Financial incentives [22,25]
	▪ Financial incentives [23,33,39,52]		▪ Alternative treatment options [27]	
**Tools**	▪ Information management system [18,19,25,30,32,33,35,39],[41,44,54,55]	▪ Standards and guidelines (equipment, practices, data) [22-27,33-35,40,44,52,56]	▪ Performance or information management system [22,24,58-61]	▪ Wait time information system [22-24,61,63]
	▪ Training and support [24-27,31,39,55]	▪ Training in WTM [17,18]	▪ Training and support of human resources [55]	▪ Standardized data [58]
		▪ Tools of communication [22,23,25]		▪ Public websites [22]

### Implementation phase of WTMS: local-level factors

#### Governance

Leadership, related to governance, was the most frequently cited factor, found in 11 articles [17-27]. The other recurrent factor, seen in five articles [19,20,24,28,29], was the establishment of a dedicated decision-making and management structure to design and implement WTMS. Finally, accountability was also an important factor in four articles, two of which identified clinician accountability as key in implementing a successful strategy [22,28], while three stressed the accountability of hospital boards and management [22,23,25].

#### Culture

Definitely the most recurrent cultural factors in the articles were related to physicians’ involvement and attitudes toward WTMS. Of the 13 articles that mentioned this factor [17,18,26,30-39], seven stressed the fact that it can be a major barrier for WTMS implementation [18,26,30,32,33,36,38]. Lack of trust between managers and clinicians was cited as another important factor [17,20,22,25,40] that can have a negative impact. On a more positive note, some authors pointed out that WTMS implementation is also facilitated by an organizational culture of quality improvement [24-28].

#### Resources

The most common local-level resource factors mentioned in the literature were those having to do with flexibility and adequacy. Insufficient infrastructure resources were mentioned as a limiting factor in several articles [17,18,20,31,37,40-44]. Capacity constraints, in relation to both operating rooms and post-surgery beds, hindered WTMS implementation [41]. One study reported that surgical capacity had to be increased to meet wait time guarantees [40]. Concerning human resources, many articles underscored the importance of having dedicated staffing. More specifically, several articles identified appropriate levels of dedicated staffing as a success factor for WTMS [17,19,20,22,23,31,41,42],[44-51], and some reported on the positive impact of using non-medical staff (physician assistants, nurse practitioners, anesthesia assistants) on reducing wait times [24-26]. Concerning financial resources, some articles cited incentives at the individual or the unit level as contributing to successful outcomes. In Ontario, offering financial incentives for physicians to use tools specially developed for assigning priorities to surgical patients seemed to have a positive impact on reducing wait times [23,39]. Disincentives were also powerful, as in Sweden, where hospitals that did not meet wait time guarantees ran the risk of being forced to send patients elsewhere at the hospitals’ expense [33,52].

#### Tools

The most commonly cited tool was information management systems designed to meet the high information demands in WTMS initiatives. Examples included databases for recording information [18,19,25,30,31,39,41,44],[53,54] and scheduling software [53]. Interestingly, it was noted that overly complex systems constituted barriers to WTMS implementation [31]. Thus, simple, user-friendly, and effective solutions for information presentation were lauded, as was system flexibility in the case of information systems implemented across a region or country [31,55]. It was also important that those concerned be able to access relevant data easily [31] and that the data be ‘clean’ [33].

Training and support were another important tool cited. The increased use of quantitative information made it important for staff to be trained to analyze basic statistics and time series data, and to acquire a basic understanding of spreadsheets [31,55]. Some articles pointed out the key role of peri-operative coaching teams in helping hospitals improve both clinical and organizational processes/practices for wait time management [24-27,39].

### Implementation phase of WTMS: contextual-level factors

#### Governance

The first contextual-level governance factor highlighted by the systematic review was the need for high-level (*i.e*., central) coordinating, reporting, and monitoring structures [17,21,23-27,32,35,37,44,54]. Several articles mentioned the need for such structures to provide guidelines and advice. Structures such as the UK’s National Patients’ Access Team [17] and Healthcare Commission [54] or Ontario’s Critical Care Expert Panel [26] positively affected outcomes at the organizational level. For example, the Critical Care Expert Panel provided advice on ‘methods to support a well-functioning critical care system that will minimize surgical delays’ [26]. Reporting was also cited as a success factor [25,32]; this refers to the healthcare organization’s obligation to monitor and present its data to the Ministry of Health or to an independent structure in charge of monitoring wait time. In particular, one article pointed out that, in Ontario, reports issued by the Ministry of Health on WTMS achievements [25] offer a certain degree of accountability to different bodies representing patients and more generally to the population. Still, those authors suggest that communication efforts should go further, providing more targeted information to educate the various stakeholders: boards, administrators, clinicians, and hospital staff [25].

Another positive contextual-level government factor identified in the literature was the involvement of stakeholders, including professional associations [22,25,26], partners such as national or provincial health ministries [22,24-27,54], patients, and other stakeholders in the health network [22,56]. Methods for involving stakeholders, which included soliciting them for information on data [37], advice on existing processes, ideas for improvements [25,26,32], and recommendations [24,26], as well as ensuring systems for reporting [37], were seen as helpful for WTMS implementation.

Another factor was related to accountability agreements. As highlighted in several articles [22-25,44,54], accountability agreements signed between government and hospital boards are a key element of Ontario’s wait time strategy. These agreements identify the hospital actors and make them accountable for equitable access to services in their organization.

Leadership was also identified as a key factor in WTMS implementation. Several articles outlined the role of provincial and regional leaders in the positive development of WTMS [23-27].

#### Culture

One cultural factor seen at the contextual level was consultation with front-line actors. This factor surfaced as having contributed to the successful implementation of WTMS initiatives [17,22-27,32,34,36,37]. An example of this was consultation with expert panels as part of Ontario’s Wait Time Strategy. Indeed, a key element of the strategy involved seeking front-line actors’ (clinicians, administrators, and researchers) advice on improving access [22-27]. These consultations helped to involve experts in the strategy and to enlist their support and involvement.

Public awareness was also identified as a success factor in WTMS implementation. Several articles described government efforts to make available to patients clear information about wait time strategies [22,23,25,39,54]. These initiatives empowered patients by sharing knowledge about wait times [25].

Developing a performance culture was also an important factor identified in the literature [22-25,27,54]. Common levers used by the Ministry of Health to create a performance culture included introducing financial incentives for physicians, monitoring data, and conducting assessments, audits, and hospital surveys, all with the aim of improving practices/process efficiency and effectiveness.

#### Resources

Funding was by far the most recurrent positive contextual resource factor [17,18,22-27,30,32,34,35,37],[40,45,48,49,52-54]. Higher level funding was provided for specific initiatives, such as strategies to address backlog [25,26,30,32,40,49], initiatives to make practices more efficient and effective [23,25], recruitment of medical staff [54], and specific purchases, such as information systems equipment for data collection [22,23,25] and new or updated medical equipment [22,23,25,54].

Financial incentives based on performance were another contextual resource factor described in some articles [22,23,25,26]. For example, in Ontario, hospitals were expected to meet a series of conditions to obtain funding for wait time cases.

#### Tools

At the contextual level, tools included instruments or procedures that affected more than one organization, such as the development and implementation of standards and guidelines [22-27,33-35,40,44,52,56]. Some procedures involved standardizing equipment across hospitals (*e.g*. MRI, CT scanners) [25,26]. Others were related to standardization of practices [23,25,26]. Standardized wait time indicators were also identified as a contextual level tool [23-27]. Finally, collection and standardization of data was the most recurrent factor cited [22,23,25,26,33-35]. Implementation of a central registry was also seen as a tool to significantly reduce wait times [33-35,37].

Training programs, whether in specific professional skills or management skills, were also mentioned in a few articles as a useful tool for implementing WTMS [17,18]. Finally, the development of communication tools such as websites was also cited in some articles [22,23,25]. For example, in Ontario, the implementation of a website made it possible to provide the population, first, with general information on the province’s wait time strategy and, later, on specific medical specialties as part of the strategy.

### Sustainability phase of WTMS: local-level factors

#### Governance

Accountability was the factor most frequently cited as a key component of WTMS sustainability [22,57,58]. Those working at the local level pointed out that accountabilities have to be explicit and must apply to all local stakeholders, including the general public and patients, healthcare providers, administrators, managers, and government. One article emphasized that each of these groups has a critical role to play in furthering the strategy’s momentum and developing a sustained culture for the future [22].

Leadership was also cited in some articles as an important factor in sustaining a WTMS [22,24,57], with the point being made that leadership must be continuously provided by all local champions, such as boards of hospitals and local health organizations, hospital administrations, and medical management.

#### Culture

Physician involvement was cited as a cultural factor influencing WTMS sustainability [22,54]. According to these articles, it is important that physicians maintain momentum in meeting wait time accountability targets. One article described an open management style that supported physicians and engaged them in activities to improve quality and access [54]. Finally, the need for cooperation between physicians and managers was also mentioned [22].

#### Resources

Certain human resources issues that pose significant challenges to improving system capacity were identified in one article [24]. The authors stressed that, to ensure WTMS sustainability, hospitals must develop effective recruitment and retention strategies in the relevant professions, where there have often been long-standing issues regarding supply. Wait time issues cannot be solved by improving clinical efficiency alone; any solution must also incorporate new ways of working. One article, for instance, described an innovative initiative to expand the role of physiotherapists in joint replacement programs [24].

Finally, the need to provide patients with alternative treatment options was presented as a key dimension of sustainable WTMS [27].

#### Tools

At the organizational level, the tool most frequently cited was the use of a performance or information management system [22,24,58-61]. These articles pointed out hospital managers’ need for appropriate information to assess their wait time performance, as well as tools for managing capacity, booking patients, and mapping peri-operative processes in order to predict maximum patient flow.

Finally, training and support of human resources was another factor presented in the literature [55]. In particular, this article mentioned the positive role of training staff in the use of common software and in data management.

### Sustainability phase of WTMS: contextual-level factors

#### Governance

Leadership was the most important contextual governance factor cited [22,24,57,58,62]. As was pointed out in several articles, leadership at the higher levels is important to sustain a WTMS.

Stakeholder involvement was another key factor [22,27,62] in sustaining WTMS, as were quality and safety measures, processes, and monitoring [54,57].

#### Culture

Some articles considered an informed public to be an important factor in sustainability [22,27]. Indeed, these articles highlighted the media’s important role in educating the public by communicating the content of public wait time websites, informing the public on how to use wait time information to discuss treatment options with care providers, and writing about the impact that consumer choices, behaviors, and expectations have on wait time.

#### Resources

Financial incentives were identified as a key factor in sustaining WTMS [22,25]. These articles highlighted the need to align hospital and physician funding mechanisms, since their different methods of payment represented a barrier to the sustainability of such strategies. Indeed, fee-for-service remuneration motivates physicians to treat as many patients as possible, whereas hospitals receiving global budgets are motivated to treat as few patients as possible. These articles outlined the need to align physician remuneration with government objectives to sustain wait time strategies. Performance-based payment was seen as an incentive to perform more procedures as efficiently and effectively as possible. Finally, the articles highlighted the need for appropriate funding for these measures [27,62].

#### Tools

Electronic information systems were cited as a key to sustaining wait time initiatives [22-24,61,63]. Indeed, the availability of wait time information was seen as enabling decision-makers to assess the impact of investments in WTMS on access and on efficiency of service delivery. Another article also highlighted standardization of wait time data as a key sustainability factor [58].

Public websites were cited as important tools for sustaining WTMS. Indeed, in some cases, communicating to the public about wait times included providing information not only on wait times for scheduled procedures but also on wait times for surgeons practicing such procedures [22].

Table 3 summarizes all the factors cited.

#### Workshop on WTMS

In March 2009, we organized a workshop that brought together 20 experts representing a cross-section of Canadian healthcare opinion and thought leaders. Participants were selected who had system and organizational level responsibility for healthcare provision, WTM leadership and/or health services research and education, and were in a position to provide experience-based critiques of WTMS. Participants included care providers, health system managers, policy-makers, and representatives of national associations of healthcare professionals and accreditation bodies. More recently, in a workshop at the Taming of the Queue conference in 2013, we were able to present and once again validate the results from our overall study.

The objectives of the 2009 workshop were to:

1. Review findings from our systematic review with an expert group of individuals who could potentially apply the research results;

2. Validate research findings with experts from different domains to ensure that the findings were sound and to explore how identification of the various factors could be developed into best practices;

3. Discuss ways to disseminate the research findings so as to be most helpful to policy-makers and managers;

4. Solicit suggestions for future research opportunities and research settings.

Workshop participants were asked to compare their experiences of WTMS and to identify success factors and constraints. Participants listed constraints and barriers to WTMS implementation and sustainability and ranked them in order of severity. Then they identified factors that improve WTMS implementation and sustainability. From this workshop, five main themes emerged. First, these healthcare managers and decision-makers had more to say about negative factors, namely constraints and barriers to WTMS, than about positive or success factors. Second, workshop participants brought forward new factors not identified in the literature that were specific to the Canadian context, particularly with regard to WTMS sustainability (Table 4). Third, they reported the same main factors for WTMS implementation as those identified in our systematic review, including leadership solutions at both the local and contextual levels (governance factors), payment incentives for physicians at the local level and funding at the contextual level (resource factors), change management support tools at the local level and standardized data at a higher level (tool factors). Table 4 presents the key barriers to both implementation and sustainability of WTMS initiatives. Fourth, they identified 10 main factors that improve WTMS implementation and sustainability (Table 5). This helped us to conceptualize, build, and test a tool to help managers implement and sustain WTMS at the organizational level. Finally, they stressed the fact that WTMS initiatives had been implemented across Canada ever since the September 2004 meeting of Canada’s First Ministers, and therefore by that time (2009) they had hoped to be seeing more studies on sustainability. Given that the first period covered by our systematic review ended in 2005, this last observation prompted us to extend our review in hopes of finding data on sustainability beyond that point. Ultimately, however, the data uncovered in the second portion of the systematic review did not really satisfy those expectations.

**Table 4:** Barriers and constraints related to implementation and sustainability of WTMS experienced by workshop participants (new factors in bold)

	**Implementation**		**Sustainability**	
	**Local level**	**Contextual level**	**Local level**	**Contextual level**
**Governance**	**• Lack of coordination among provider groups**	**• Limited information sharing**	**• Lack of management of critical diagnostic, surgical and continuity of care interdependencies**	**• Limited information sharing**
	• **Limited information sharing**	**• Competing health system priorities**	**• Lack of time allotted to WTM strategies**	**• Competing health system priorities**
	• Lack of leadership for solutions	• Lack of leadership for solutions	**• Limited information sharing**	**• Lack of shared learning opportunities among jurisdictions**
			**• Lack of clinical-administrative partnerships**	
**Culture**	**• Competing cultures**	**• Lack of systematic approach to implement culture change**	**• Prevalence of a ‘blitz mentality’ among healthcare innovators**	--------
	**• Lack of evidence about value of WTM strategies**	• Resistance to change and uncertainty		
	• Resistance to change and uncertainty			
**Resources**	• Lack of incentives (physician payment systems)	• Incentives (physician payment systems)	--------	• Lack of funding
	• Insufficient number of administrative staff	• Lack of funding		
**Tools**	• Poor resourcing of technology and staff	• Lack of standardized data	---------	---------
	• Lack of change management support and tools			

**Table 5:** Workshop participants’ 10 best strategies and practices to improve implementation of WTMS and help sustain their success

**Best strategies**	**Practices**
**1. Greater alignment**	Align agendas across healthcare organizations; focus on the patient.
**2. Increased and strategic communications**	Increase communications among stakeholders, communicating at the right place and time and to the different levels of responsibilities.
**3. Strong data**	Establish a strong wait time management (WTM) data repository and ensure WTM data standardization; collect data about the impact of WTMS and identify gaps and goals; note that WTMS projects need to include change management, and that people, processes, and flows must be addressed.
**4. Clinical and administrative champion-partners**	Clinical and administrative WTMS champions must form a partnership; the system must identify who these champions are, define and resource their roles and actions, and enable them to implement an operational plan.
**5. Clear articulations of the value proposition for WTMS**	People involved in WTMS must feel that it is part of an integrated strategy and not a ‘stop-gap measure.’
**6. Patient engagement**	Engage and activate patients; make the current system dysfunctions transparent so that patients understand there are differences in wait times among physicians, and provide them with the option of being seen by the first available physician.
**7. Health system trade-offs and patients’ options**	Talk about what the health system is for and what the trade-offs are for immediate access.
**8. Establish incentives**	Create a system with incentives for clinicians that involves paying them for their time.
**9. Leadership**	Leadership is required in partnership with payers. Make sure the ministries of healthcare are at the table; otherwise the lack of relationship with them can become a barrier.
**10. Expectations management**	As a parallel strategy, ‘expectations management’ is recommended around WTMS potential and limitations.

## Discussion

From this systematic review of 47 articles, we were able to identify key factors influencing WTMS, especially for implementation. Few studies explored the determinants of WTMS sustainability [22-26,57-63]. With regard to evidence-based healthcare programs and practices, two recent systematic reviews looked at factors that influence the sustainability of such initiatives [64,65]. Most of the studies reviewed there did not define the notion of sustainability in clear terms, and most did not use a rigorous research design to evaluate research findings on factors impacting sustainability [65]. Although that review found the same main determinants of sustainability as emerged in our review, the factors identified were less accurate than those we were able to show. For example, that review did not identify subcategories for factors related to the cultural dimension, such as performance culture, quality improvement, or public awareness and empowerment. Furthermore, the studies reviewed did not always link the determinants of healthcare innovations’ sustainability to their impacts on particular goals or on benefits for patients.

Despite the lack of evidence in the literature on the notion of sustainability and its determinants, the discussions in the workshop helped to shed light on this notion. Indeed, the workshop allowed us to identify additional constraints and barriers related to WTMS sustainability (see Table 4). Moreover, the workshop participants’ notions concerning implementation differed from those identified in reviewed articles in two ways. First, the participants identified factors that negatively impacted WTMS implementation, in contrast to the success factors found in articles. They also identified factors, again negative, that were especially related to sustainability and were not in the literature. Most of those were related to governance at the local level, such as limited information sharing between professionals and managers, lack of time allocated to WTMS, and lack of partnership between professionals, especially physicians, and managers (see Table 4). Despite these differences, many of the factors identified in the literature and the workshop were similar, including those relating to implementation (see Table 4). As such, this process highlighted the importance of taking into account the potential limitations of using a theoretical framework for data extraction [14].

Because the workshop experts were healthcare managers and decision-makers directly involved in wait time reduction strategies in Canada, their input helped validate the findings and robustness of our systematic review, in addition to complementing our findings with sustainability factors that were rarely addressed in the literature.

We should also point out that an article published by our team in 2010 on a study of success factors in WTMS implementation showed the same main findings as did our systematic review [13]. That study used data from interviews with key physicians, managers, policy-makers, and researchers from across Canada who had worked on initiatives to reduce wait times. The same governance factors were noted in that article, as were cultural factors related to physician involvement in WTMS and the need for a quality improvement culture. That study also highlighted the importance of aligning contextual-level and local policies. Funding levels and financial incentives were addressed both in that article and in our systematic review. As well, subsequent to that 2010 article and inspired by it, a very interesting tool was developed to assess the success of WTMS implementation, which was applied in an orthopedic program [66] (see Table 6).

**Table 6:** **Checklist of factors that influence WTMS implementation**[66]**, as applied in an orthopedic program**

**Factors**	**Actions/Activities**
**High level coordinating, reporting monitoring structures**	• Advisory Committee established, including VP of Acute Care, Directors, and Provincial Wait Time Manager, monthly meetings
	• Regular reporting of progress to Regional Surgical Services Leadership Team and Acute Care Directors Committee
	• Quarterly updates provided to CEO, Board of Trustees and Ministry of Health
	• Indicator added to organizational Strategic Plan and Scorecard for three-year cycle (April 2011-2013)
**Stakeholder engagement**	• Numerous meetings and presentations to internal staff, including nurses, doctors, allied health and support staff
	• Presentation to Minister of Health
	• Briefing note/budget submitted
	• Regular meetings with Vice President
	• Meeting with past President of NL Medical Association
	• Presentations to Community Medical Advisory Committee
	• Department of Health Sponsor / meetings with Wait Time Management Coordinator
**Strong management and clinical leadership**	• Project Steering Committee established
	• Direct reporting to Vice President
	• Director & Clinical Chief of Surgery - Project Sponsors
	• Project Lead hired to support project
**Dedicated and stable decision making and management structures**	• Advisory Committee established
	• Project Team
	• Director & Clinical Chief members of Advisory Committee
**Consultation with frontline actors**	• Presentation to Orthopedic Education Days and Surgical Rounds
	• Weekly meetings with frontline stakeholders to establish algorithm for new referral practice, including clerical staff, allied health disciplines and managers
	• Monthly consultation and in-servicing to relevant program staff along the continuum
	• Standard Referral Working Group
	• Inpatient Working Group
	• Orthopedic Charge Nurses, clinical staff participating in site visits
	• Established formal orientation package for assessment by clinic staff
	• Assessment clinic education day organized to facilitate clinical skills upgrading and clinical practice review
	• Cross-site / multi-program working group
	• Meetings with surgeons’ secretaries
**Physician involvement**	• Presented at Surgical Teaching Rounds
	• Meeting with each surgeon individually
	• Physician sponsors/ champions identified
	• Developed a broad based communication strategy targeting multiple mediums to facilitate physician engagement and communicate planning including:
	✓ Visits to urban and rural family physician clinics
	✓ Family Physicians invited to participate in developing algorithm for changes to referral practices prior to development of referral tool
	✓ Anesthetists / surgeons working group
	✓ Teleconferences / site visit for anesthetist
	• Surgeon Champion appointed to establish strong leadership and obtain buy-in for Central Intake Process
	• Provincial Medical Association engagement: collaboration with the Communications team to communicate central intake information tools and updates to physicians via web-based media and provincial newsletters to membership
**Funding levels and earmarked resources**	• Budget request for Project Team 2011/2012 - approved
	• Department of Health funding for Project Lead
	• Health Canada funding obtained
**Appropriate levels of dedicated staffing**	• Increased staffing to facilitate enhanced clinical capacity for assessment clinic and to establish formal interdisciplinary case management
	• Funding secured for two-year pilot with dedicated staff
	• Project Lead - funded for additional year
	• Clerical Position allocated for data collection
**Flexible, adequate capacity**	• Orthopedic clinic space renovation: increased space for increased clinic capacity by nine half-day slots
	• Evaluation of existing clinical booking practice to redistribute patient ratios, improve efficiency, and increase capacity
	• Additional orthopedic operating room capacity assigned (34% increase including dedicated trauma time)
	• Additional inpatient bed capacity
**Individual and unit/team incentives**	• Adult Orthopedic Team - CEO Award for Team Excellence
	• ‘Improving Access’ poster presentation selected for Taming of the Queue, 2012 – Ottawa.
	• Key performance indicators collected and shared with team to support improvement
**Central Registries (the collection and standardization of data)**	• Wait Time 1 defined
	• Data fields incorporated into standardized referral tool to collect Wait Time 1
	• Central Intake Registry established
	• Orthopedic Wait List Data Value Stream Map Session: Full day event organized for all stakeholders
**Standards and guidelines**	• Development of algorithms, pathways for central intake process
	• Evaluation Framework developed
	• Guidelines for completion of standardized referral tool
	• Definitions for Wait 1 and Wait 2
**Information Management Systems**	• Represented on working group
	• Meetings with IMT representative ongoing
	• Site Visit (Holland Clinic, Toronto) for demonstration of
	• Central Intake Booking System
**Training and support**	• Site visits to Edmonton, Halifax, Toronto, and Vancouver
	• Participation in National Best Practice Initiative – Bone and Joint Canada: representation from all allied health disciplines, surgeons and medical staff.
	• Best Practice Toolkit introduced: Bone and Joint Canada coordinators invited to participate in multisite education event

Some of the factors identified in our study have been mentioned in other Canadian and international studies published in the grey literature. For example, the Canadian Centre for Policy Alternatives (CCPA) report in December 2005 [67] mentioned coordination, dedicated evaluation tools, commitment and cooperation by all stakeholders, and an innovative culture of performance as the main factors that can enhance the success of a WTMS. The March 2006 report of the Association of Canadian Academic Healthcare Organizations [68] and the June 2006 Final Report of the Federal Advisor on Wait Times [69] also mentioned some of the factors identified in our study, such as those related to physicians’ involvement in WTMS. Another publication, by the Health Council of Canada [6], reported that interviews with leaders of initiatives to reduce wait times in Canada had revealed a number of key success factors. Unsurprisingly, these are similar to the factors identified in our study and include: support from governments leaders; strong program leadership that brings together administrative and clinical champions; full-time staff who are dedicated to making the program work; information systems that enable programs to centralize wait lists, to track wait times in local areas and province-wide, and to share this information publicly; adequate funding for the introduction of information systems and effective program leadership; and a broad, comprehensive approach to the many large and small changes required to reduce wait times for care [6].

At the international level, a study conducted by the King’s Fund in the UK [70] examined organizational characteristics that sustained WTMS improvements. Once again, some factors identified in our study were also in the King’s Fund report, such as the need for reliable information, strong leadership, and incentives, although our findings were more extensive.

### Limitations of the systematic review

Our focus on the factors themselves, rather than on their impact on reducing wait times, was both a strength and a weakness. However, there are few studies on wait time reduction and on the success factors linked to WTMS impact on wait time. We did not establish a scale to measure the quality of evidence. Nor did we assess the quality of the research design for each study. Rather, we focused on our main objective, which was to identify the factors at different levels, and we used the conceptual framework to categorize each factor and to abstract all the articles in a consistent manner.

Another limitation of our review was that it included only English and French language studies and those published in peer-reviewed journals. Nevertheless, we were also able to consult the grey literature and to discuss it in the workshop and in subsequent presentations at conferences.

## Conclusion

This is the first published review on factors that can influence success in implementing or sustaining WTMS in healthcare organizations. The conceptual framework used in this study is one that we previously used for a published study on success factors of WTMS implementation, which was based on interviews with physicians, healthcare managers, and policy-makers [13]. Our framework, inspired by Parson’s widely recognized and robust four-quadrant model [12], was useful for organizing and analyzing the factors identified in the literature as influencing the implementation and sustainability of WTMS at both the systemic and local levels.

The paucity of articles with evidence on sustainability points strongly to the need for more research in that area related to impact on WTMS. Since our systematic review did not explore how these individual factors influenced reduction of wait times, future research should focus on success factors that help to both implement and sustain WTMS and that ensure reduction of wait times.

Nevertheless, our results can be useful to healthcare decision-makers and managers seeking to develop best practices based on success factors for WTMS implementation and sustainability. One example of such use is the tool presented in Table 5, which is a checklist of factors developed to support managers at the organizational level implementing a WTMS in one of the main orthopedic services of Ontario’s Eastern Regional Health Authority. This tool was shown, in a study, to have been a successful method of knowledge translation that contributed to the sustainability of that initiative [66].

## Abbreviations

WTMS: Wait time management strategies; CINAHL: Cumulative Index to Nursing and Allied Health Literature; MEDLINE: Medical Literature Analysis and Retrieval System Online.

## Competing interests

The authors affirm that they have no competing financial or non-financial interests to declare.

## Authors’ contributions

MPP was the PI responsible for leading the project. MPP, PGF, CS, CD, NC, and MD identified and analyzed the articles. MPP, MD, and NC wrote the first draft of the article. PGF, CS, CD, EW, and TN were involved in writing the final draft. All authors read and approved the final manuscript.

## Authors’ information

Marie-Pascale Pomey, MD, MSc, PhD, is an associate professor in the Department of Health Administration, Public Health Research Institute, Faculty of Medicine, University of Montreal. Pierre-Gerlier Forest, PhD, is president of the Pierre Elliott Trudeau Foundation. Claudia Sanmartin, PhD, is a senior researcher in the Health Analysis Division, Statistics Canada. Carolyn DeCoster, PhD, RN, MBA, is executive director for Clinical & Zone Analytics, Data Integration, Measurement & Reporting, Alberta Health Services. Nathalie Clavel, MHA, is a research assistant, IRSPUM. Elaine Warren, RN, MSc, is regional director of Surgical Services, Eastern Health, and program director of Critical Care, Eastern Health. Madeleine Drew, MHA, is a research assistant, IRSPUM. Tom Noseworthy, MD, PhD, is an associate chief medical officer for Strategic Clinical Networks and Clinical Care Pathways, Alberta Health Services.
